# Temporal variation in microplastic abundance in sediment, fish and shrimp: a case study in the Thames Estuary, UK

**DOI:** 10.1098/rsta.2025.0040

**Published:** 2025-10-23

**Authors:** Alexandra Rachael McGoran, Paul Frederick Clark, Brian Smith, David Morritt

**Affiliations:** ^1^ CEFAS, Lowestoft, UK; ^2^ Department of Life Science, Natural History Museum, London, UK; ^3^ School of Life Science and the Environment, Royal Holloway University of London, Egham, UK

**Keywords:** microplastics, temporal variation, plastic pollution, bioavailability

## Abstract

Microplastics are abundant in marine and terrestrial habitats, with rivers transporting particles to the sea. The River Thames catchment, UK, encompasses 15 million residents and many pollution sources. Temporal trends in microplastic abundance are sparse, with ad hoc studies in the estuary demonstrating great variability in concentrations. Taking a seasonal approach to time-series data collection, sediment and biota were sampled every three months over 2 yr. To account for the heterogeneity of microplastics in sediment, three grab samples were collected per survey. On average, 1000 ± 1100 (s.d.) plastic items kg^−1^ were recovered from sediment, with significantly more from samples in July (summer) and September (autumn) 2020. The recorded concentrations were comparable with studies worldwide. Biota in the estuary are exposed to this plastic. Fish (*Osmerus eperlanus, Platichthys flesus, Solea solea*) and shrimp (*Crangon crangon*) contained on average 1.59 ± 2.62 items per individuals and 0.39 ± 0.95 items per individual, respectively. The greatest proportion of contaminated individuals was in December 2018 (winter; 75%) followed by March and June 2019 (spring; 42% and summer; 43%, respectively). Seasonal factors, such as rainfall, can affect plastic accumulation in an estuarine system, but these microplastics are not always bioavailable. Understanding the drivers of this variability is key in designing mitigation strategies and managing risk.

This article is part of the Theo Murphy meeting issue ‘Sedimentology of plastics: state of the art and future directions’.

## 1. Introduction

Plastic pollution is a ubiquitous, global issue [1]. In the environment, large plastic items fragment into smaller fractions [2–5], which, along with primary microplastics such as microbeads, are widely bioavailable and pose a risk to biota [6,7]. Ingested microplastics can cause physical damage to the digestive tract [8], reduce feeding rates [9–11] and may lead to false satiation if present in high numbers [12]. They also adsorb and leach persistent organic pollutants and plastic additives [13–15] and can act as vectors for disease [16,17] and biological toxins [18]. There is increasing evidence that co-exposure with microplastics increases the harm caused by other environmental stressors, such as pesticides [19], bacteria [20] and harmful algal blooms [21]. These microplastics (smaller than 5 mm) are abundant in air [22], water [23,24], sediment [23,25,26] and biota [23,26–28]. Microplastics historically encompassed traditional crude-oil derived polymers, such as polypropylene and polyethylene. With the development of biodegradable plastics and modified semi-synthetic materials this categorization becomes more difficult. The term ‘microlitter’ can be used to cover all anthropogenic materials including plastic, rubber and semi-synthetic materials. OSPAR, however, have included rayon, a semi-synthetic cellulosic material, as essential reporting under microplastics [29]. As such, the present study refers to items collected as plastics, encompassing meso- and microplastics, throughout. While evidence for the abundance of microplastics in the environment is mounting there is still a requirement to understand the transport and sources of this pollution, as well as its risk to biota.

Rivers are a key source of plastic to the marine environment, transporting waste from land-based sources such as agriculture, roads and urban centres [30–35]. As the field of microplastics research has increased, so has the number of studies into estuaries, but sinks and sources in these systems have been highlighted as a knowledge gap [36]. Seasonal assessments of the occurrence of this plastic are key to providing data for accurate modelling of transport mechanisms. Studies on this topic are, however, scarce. Windsor *et al.* [37], for instance, has highlighted a lack of understanding in residence time of riverine plastics. Furthermore, monitoring environmental microplastic concentrations is key to quantifying risk and evaluating the success of mitigation strategies.

Estuaries are essential habitats for many species and the abundance of microplastics in these environments can have severe effects on ecosystem health. Microplastics are widely bioavailable and are thus readily ingested. Microplastic ingestion can negatively affect growth, immune response, food consumption, fecundity and energy levels as well as having generational effects [38,39]. Several studies report ingestion of microplastics, often investigating single species and not exploring links between risk and the organism’s ecology. Further study is required to understand the effect of habitat, trophic level, feeding strategy, diet, behaviour on microplastic ingestion and retention [40]. Thus, studying estuarine biota along with environmental compartments over a time series addresses several knowledge gaps.

While generally well studied [41–52], the River Thames still encounters the same research challenges as those highlighted by Danopoulos *et al.* [53] (e.g. collecting representative samples, lack of funding for long-term studies). With the depth of knowledge on microplastics in the estuary it makes a suitable site from which to perform case studies to address knowledge gaps in the field, such as the volume of meso- and microplastics retained within riverine system, the variation in this retention throughout the year, whether environmental factors such as rainfall affect retention in sediment and if varied plastic loading affects the bioavailable fraction of plastic. The aims of the present study were to (i) address seasonal variation in microplastic abundance and ingestion; (ii) assess the link between rainfall and microplastic abundance in the Thames Estuary.

## 2. Material and methods

### (a) Study site

**Figure 1.**
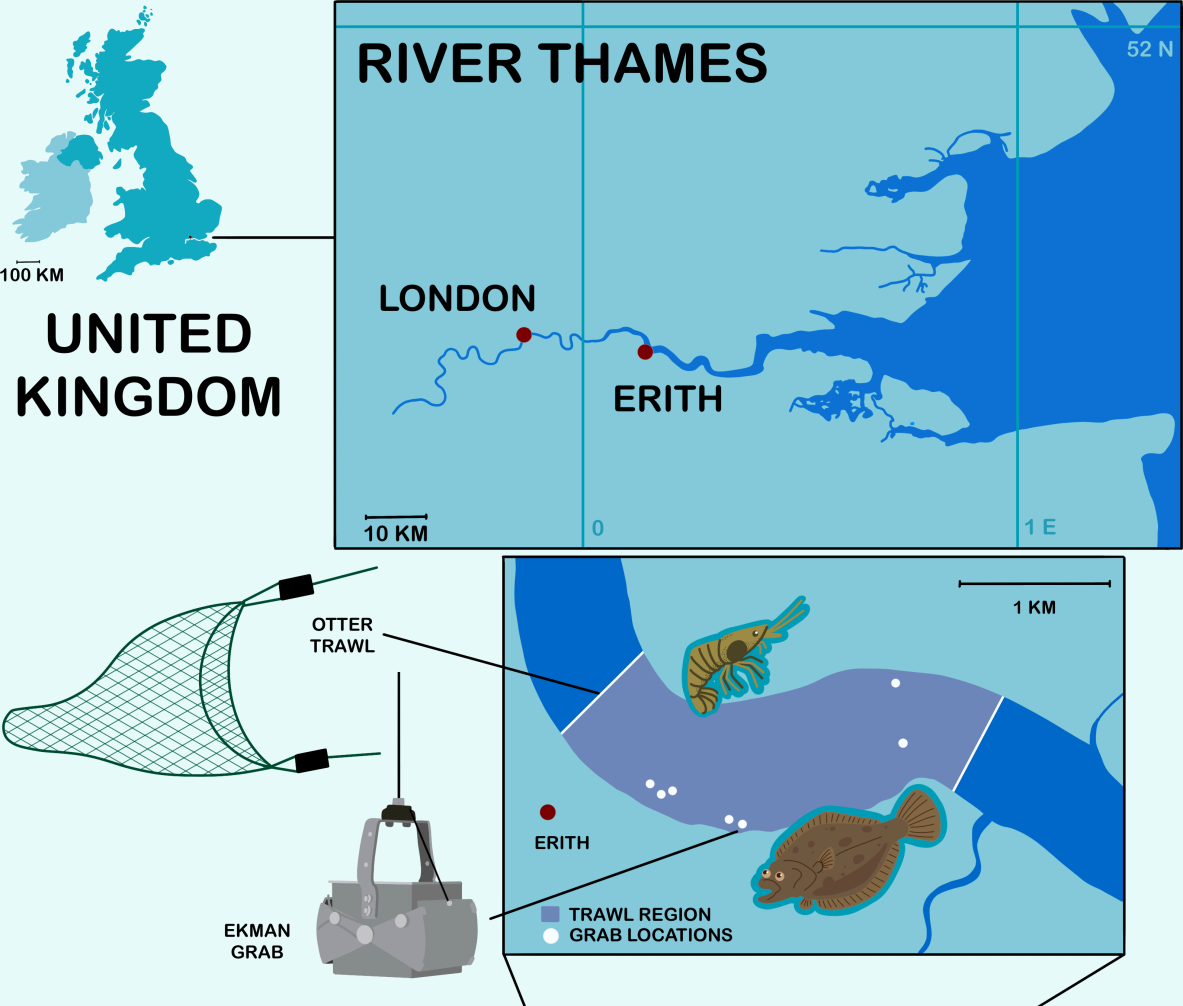
River Thames sampling sites. White dots mark the location of sediment grab samples, with the region sampled by trawl marked between the white lines.

The River Thames is the second largest river in the UK and is situated in South East England (figure 1). It has a catchment of 16 000 km^2^ which is primarily rural, with 17% urbanized mostly in the east [54,55]. The estuarine reach of the Thames is turbid and highly tidal [56] with average flow rates of up to 78.17 m^3^s^−1^ at Kingston, south-west London. The mean annual rainfall in the catchment is 710 mm [57] and freshwater entering the estuary has an average residence time of two months [56].

In the UK, summer starts in June and ends in August. It is the hottest and driest part of the year, but rainfall varies considerably in summer with several floods occurring. During autumn, temperatures reduce and storms increase. Winter begins in December, it is the coldest season, has the shortest days, and can be variable, with some years experiencing several storms and strong winds. Other years the temperature decreases more with fog, frost and snow persisting. As spring starts in March, days begin to get longer and the temperature increases. Spring is usually a calm and dry season [58].

### (b) Quality assurance and contamination controls

In the field the Ekman grab and sample storage items were kept closed. Boxes and bags for sample storage were additionally stored in a closed cool box until use, minimizing airborne contamination. These were also rinsed prior to packing. While items were plastic, rinsing of items before use was considered to remove any potential contamination before they were used for sample storage.

In the field, all containers were kept covered and enclosed in a cool box to prevent airborne contamination. Containers were cleaned with filtered water prior to packing. The lids for containers were only removed for the minimum necessary time to fill with sediment (an approximate maximum of 1 min). All laboratory work was conducted under a laminar flow cabinet in a clean laboratory with sticky contamination control mats at the entrance. Only 100% cotton clothing and purple 100% cotton laboratory coats were permitted in the clean laboratory. All doors were kept shut to reduce airborne contamination from adjoining spaces. All solutions were filtered through a 32 μm mesh (distilled water was run through a metal sieve, potassium hydroxide (KOH) and ZnCl_2_ were vacuum filtered through nylon mesh). Equipment and glassware were triple rinsed with filtered distilled water. For all work nitrile gloves were worn. Biota were rinsed with filtered water prior to dissection. Laboratory blanks (negative controls) were collected during sample processing. These were processed identically to samples. In addition, empty Petri dishes were used to record airborne contamination during dissections, digestions, filtration and visual examination. Potential sources of contamination highlighted by these measures were removed from the laboratory. For instance, initial controls showed a high abundance of white fibres which could originate from many sources. To better identify contamination in samples, lab coats were dyed purple and all white lab coats were removed as were white mesh curtains at the entrance to the lab. Sponges were removed from the lab as they could shed microplastics from the abrasive surface. All samples remained covered, whenever possible, including in the oven, during filtration, digestion and density separation. Filtered samples were covered by Petri-dish lids and sediment-microplastic isolation units were covered with foil. Contamination in procedural blanks was removed from samples on a particle-by-particle basis. The colour and shape of recorded contamination was used to remove specific items from the results, i.e. the average number of clear fibres recovered in controls were removed from samples.

### (c) Sample collection

As part of an ecosystem-wide approach to monitoring plastic across size ranges (micro to macro) [59], all samples were collected from Erith during one survey programme from December 2018 to September 2020. Permission to trawl and collect samples in the Thames Estuary was granted by the Port of London Authority, Marine Management Organisation and the Environment Agency. Catch limits were established and adhered to during sample collection. If additional sampling resulted in the excess of catch of species already at their quota (20–30 individuals per fish species), trawling was suspended. Dead individuals were permitted to be kept; thus maximum samples were *n* = 50. The programme consisted of eight surveys *ca* three months apart. Trawls and sediment grabs were utilized on all surveys. An Ekman grab was deployed from the vessel at slack tide at a depth of *ca* 12 m. The grab was deployed three times to collect triplicate 3 l samples. Sediment, covered by estuarine water, was stored in a sealed plastic container at room temperature until analysis (*ca* a few days to a few months).

To collect biota, an otter trawl with a mouth 10 m by 1 m and mesh size of 80 mm with 16 mm insert to ensure the collection of shrimp was deployed from a fishing vessel *Boy Daniel SD4*. On each survey date up to five mid-water column and five riverbed (*ca* 9−12 m) 15 min trawls were conducted near the banks of the Thames. Midwater and benthic trawls were conducted in approximately the same location (electronic supplementary material, table S1). A wide range of biota were collected. For the present study, only species that were abundant on all sampling occasions were investigated: *Platichthys flesus,* European flounder; *Osmerus eperlanus,* European smelt; *Solea solea,* Dover sole; *Crangon crangon,* brown shrimp. Biota were stored on ice overnight until they could be frozen the following morning at −20°C.

### (d) Sample processing and analysis

Plastics were extracted from sediment following the method of Coppock *et al.* [60]. Samples were dried at 50°C in an oven for 24 h or until dry. From each of the three grab samples five replicates of 50 g dry weight (*ca* 50 ml) were collected. Exceptions were made for sediment samples collected on 19th December 2019. Due to laboratory access restrictions with the COVID-19 pandemic it was only possible to collect two subsamples from one of the grabs, resulting in 12 replicates instead of the 15. Each 50 g replicate was suspended in 700 ml of filtered 1.5 g cm^−3^ zinc chloride (ZnCl_2_) solution. The sample was agitated for 5 min with a magnetic stirrer, followed by a 5 min rest period. Three rapid pulses of the magnetic stirrer were then used to displace air bubbles. The solution was allowed to settle until no sediment was suspended in the solution. This took from 15 min to 7 h. When all the sediment had settled, the ball valve was closed, the solution filtered through a 32 µm white nylon filter with a vacuum pump and the filter subsequently dried at 50°C in glass Petri dishes in an oven.

Biota were defrosted prior to dissection. The mass (Ohaus Ranger 3000 balance, nearest 0.1 g), length (to nearest mm) and sex of each individual was recorded. For fish, the entire digestive tract and the gills were recovered and each was placed in a separate 50 ml centrifuge tube (Falcon). Shrimp were cut in half dorsoventrally to expose the gastrointestinal tract and gills and then individually stored in 50 ml tubes. The tubes were filled with 10% prefiltered (32 µm) KOH solution so that all tissue was covered and were incubated at 50°C. Following overnight digestion, samples were filtered through a 32 µm nylon filter with a vacuum pump. Tubes were further triple rinsed with clean distilled water (filtered through 32 µm metal sieve) to ensure all material was recovered. Filters were dried at 50°C in glass Petri dishes.

Visual examination was conducted under 16−64× magnification with a Leica MZ 6 dissection microscope with reference to Lusher *et al.* [61]. The detection limit was *ca* 32 µm due to the pore size of the filters used. All recovered items were photographed and described by colour and shape. Shape was classified as either a film, fibre, fragment or a tangle of fibres. In some instances, items did not fit these criteria and were listed as ‘other’. This category contained three resin-like, putties that do not fit within current classification [62].

A representative 10% subset was analysed by Fourier-transform infrared spectroscopy (FTIR) and compared with commercial and in-house spectral libraries [59], with their length and width recorded using Image J version 1.52 a [63]. The subset was selected by analysing a random 10% of all colour and shape combinations (e.g. red fibres, blue beads) for sediment and each analysed species separately. Two FTIR instruments with identical libraries were used for infrared analysis of plastics. It was not possible to use one instrument for the total duration of the study due to damage to the first instrument, resulting in its replacement. A PerkinElmer Spectrum One FTIR spectrometer, with an AutoIMAGE FTIR Microscope System PerkinElmer attachment was initially used alongside OMNIC Picta. Individual items from the subset of plastics were individually analysed with a background spectrum collected before analysis of every item. A total of 16 scans between 500 and 4000 cm^−1^ were collected per analysis. Later samples were analysed with a Nicolet iN10 MX Infrared Microscope in OMNIC Picta. Absorption spectra were collected with an MCT-A detector over 16 scans at a resolution of 4 cm^−1^ in the range 650−4000 cm^−1^. Polymer identity was confirmed by visual comparison with library spectra (electronic supplementary material, table S2) rather than with a percentage match threshold.

### (e) Statistical analysis

Statistical analysis was conducted in R version 4.0.3 [64] with the following packages: ‘ggplot2’ [65], ‘MASS’ [66] and ‘multcomp’ [67].

Daily rainfall data collected by the Met Office [68] were accessed through the Centre for Environmental Data Analysis. The closest active station to the sampling site was Dartford S WKS. Only rainfall recordings 7 d prior to sampling and the day of sampling were analysed. Shapiro–Wilk tests were used to determine whether plastic abundance in sediment and biota and cumulative rainfall 7 d prior to sampling were normally distributed. Following this, a correlation test (Spearman’s rank) was performed between plastic abundance and rainfall for both matrices (see electronic supplementary material item 3 for R Script). The same approach of a Shapiro–Wilk test followed by Spearman’s rank test was used to determine whether rainfall (both cumulative and single rainfall the day prior to sampling) correlated with the diversity of plastic present in sediment samples. Diversity was measured as the average number of shape and colour combinations, e.g. white fibre, green bead, black fragment.

Generalized linear models (GLMs) were used to compare the amount of plastic and proportion of affected individuals across sampling dates. The best fit model was determined by comparison of Akaike information criterion scores with consideration for overdispersion and residual distribution. Poisson, negative binomial and gamma distributions were tested for fit; R code and plots are available in the electronic supplementary material. Non-significant variables were systematically removed until only significant factors remained. Significant variables were then analysed with a pair-wise Tukey post hoc test. Due to the high proportion of biota that typically do not ingest plastics, datasets are often skewed and cannot be transformed due to the nature of count data. To account for the positive skew in the data, zero values were removed from the biota dataset for statistical analysis of microplastic abundance. Zeros remained for analysis of the proportion of contaminated individuals.

Model 1 – GLM (plastic per gram sediment ~ date, family = gamma (link = log))

Model 2 – GLM (proportion to ingest plastics ~ date + species, family = binomial)

Model 3 – GLM (ingested plastic abundance ~ date + species + length + mass, family = gamma (link = inverse))

## 3. Results

### (a) Environmental conditions

**Figure 2.**
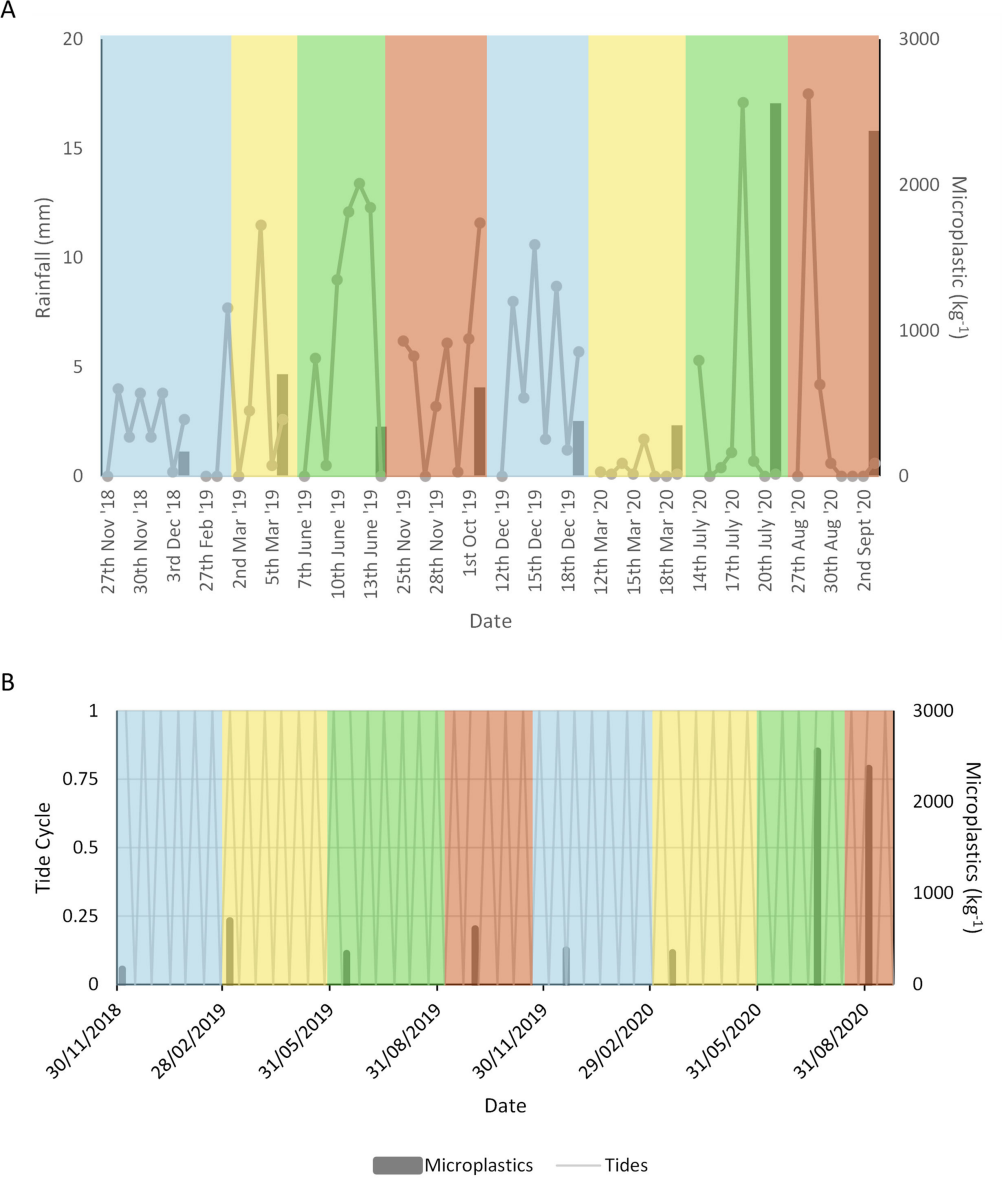
Mean plastic abundance in sediment kg^−1^ (secondary *y*-axis) plotted against (A) daily rainfall [47] for the week leading up to sampling (primary *y*-axis); (B) spring-neap tide cycle where 1 is a spring tide and 0 represents a neap tide. Seasons are represented by colours with (in order of appearance left to right) blue for winter, yellow for spring, green for summer and orange for autumn.

The average rainfall prior to sampling was 28.13 ± 15.33 mm. Cumulative rainfall was greatest in June 2019 at 52.7 mm (figure 2A). The greatest rainfall on a single day occurred in July and August 2020 with 17.1 mm and 17.5 mm, respectively. Spring-neap tidal cycles are shown in figure 2B. Three of the eight samples were collected at or close to spring tides, with the remaining samples collected closer to neap tides.

### (b) Contamination

Sediment processing controls included clear fibres, clear films, blue fibres, black fibres, red fibres, purple fibres, green fibres, grey fibres, yellow fibres, white fibres and white films. On average 3.8 microplastics from each replicate were removed as contamination. Biota processing controls contained clear fibres, clear films, blue fibres, blue films, blue fragments, black fibres, black films, black fragments, brown fibres, red fibres, red films, white fibres, white films, grey fibres, green fibres, green films, green fragments, yellow fibres, pink fibres, purple fibres, purple fragments and tangled fibres. Samples were corrected on average by 1.6 items per sample.

### (c) Sediment

**Figure 3.**
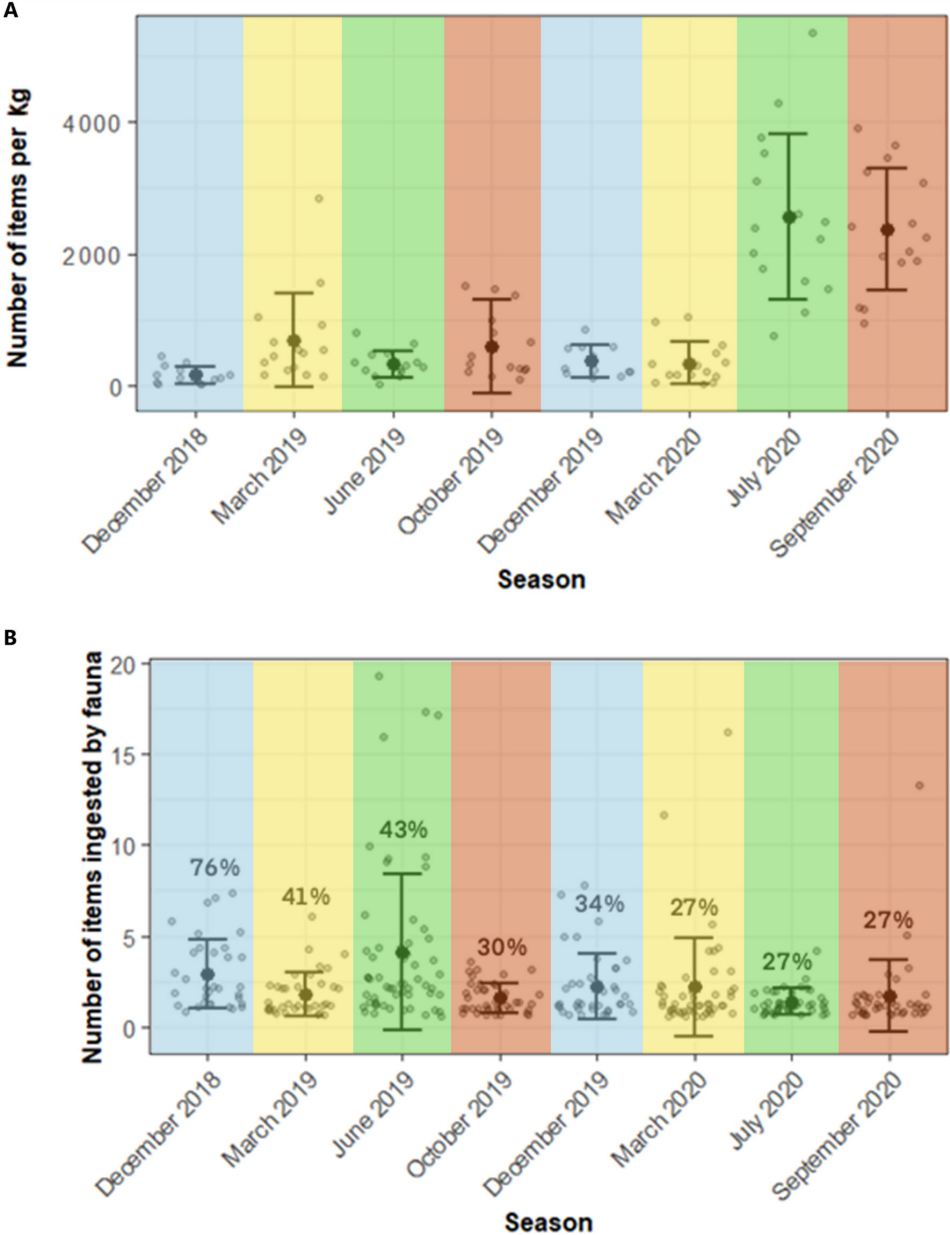
Plastic abundance per sampling date in (A) sediment and (B) biota with percentage of contaminated individuals above. Grey points are individual values, black points are the means and bars are ± s.d. Seasons are represented by colours with (in order of appearance left to right) blue for winter, yellow for spring, green for summer and orange for autumn.

**Figure 4.**
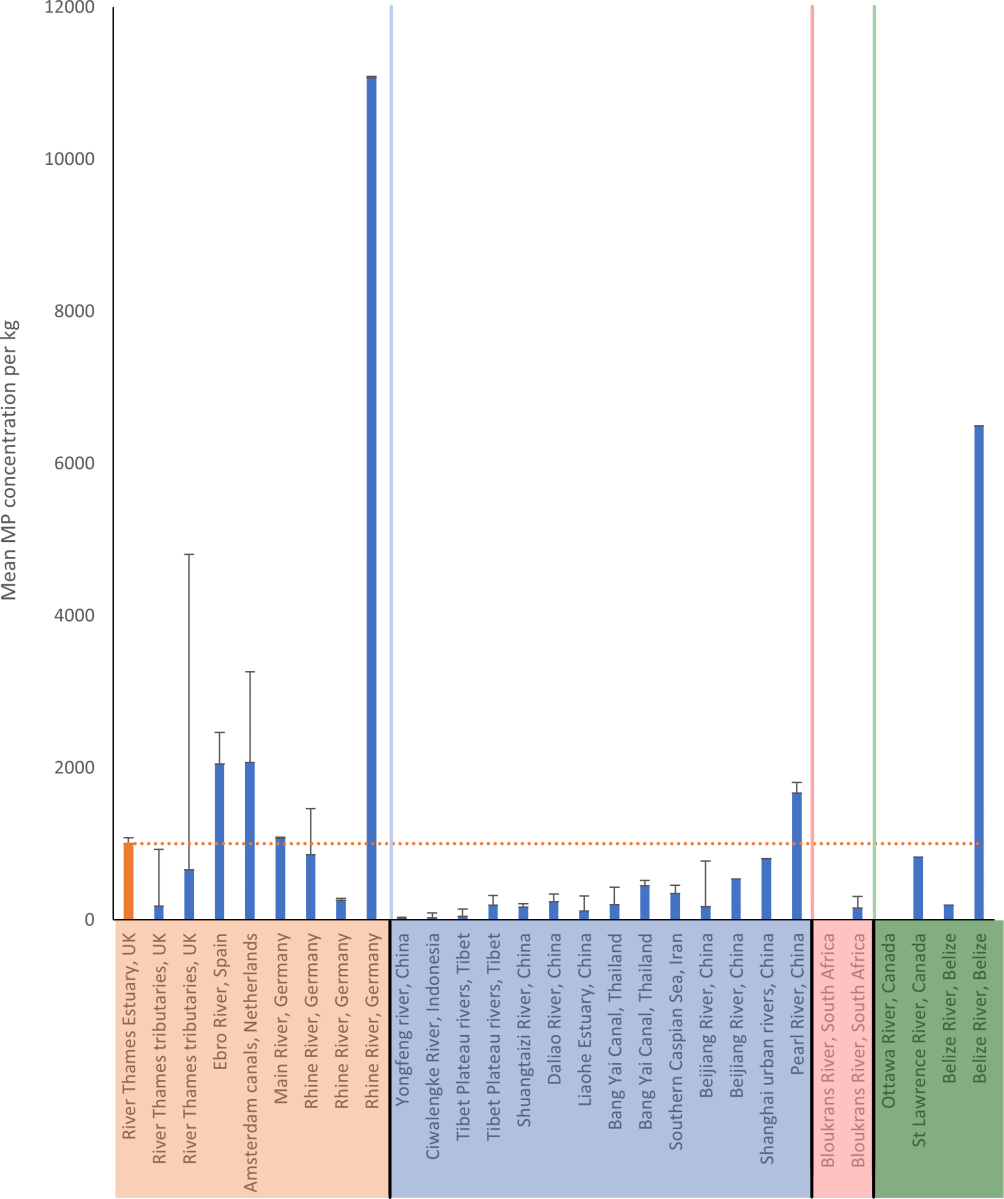
Global mean plastic concentrations in riverine and estuarine sediments (kg^-1^). Locations are highlighted by region, (in order of appearance left to right) orange are concentrations from Europe, blue from Asia, red from Africa and green from the Americas. Error bars are ± s.d. More details can be found in the electronic supplementary material, table S4.

All sediment samples contained plastic items with 5557 items recovered overall. On average (± s.d.) 1000 ± 1100 items kg^−1^ were present in the sediment (figure 3A), with concentrations ranging from 170 ± 130 items kg^−1^ (winter; December 2018) and 2560 ± 1250 items kg^−1^ (summer; July 2020). A comparison with global estimates of microplastic abundance is recorded in figure 4 with references available in the electronic supplementary material, table S4. In the present study, particle size ranged from 94 µm to 43 mm. The average length of plastics in sediment ranged from 1.4 ± 1 mm in December 2019 and 3.2 ± 6.0 mm in March 2020 (electronic supplementary material, table S5). Only 5% of items were considered mesoplastics (electronic supplementary material, fig. S1). Clear and white fibres were the predominant form of plastic in sediment for all sampling seasons (28–43%). Black and red fibres were also abundant for all sampling dates, in the ranges 4−12% and 0−8%, respectively. Blue fibres were common in March 2019 (spring, 3%), July 2020 (summer, 4%) and September 2020 (autumn, 4%).

In Model 1, sampling date was significant (*p* < 0.05) with sediment samples from July 2020 (summer) and September 2020 (autumn) being significantly more contaminated on average (2600 ± 1300 and 2400 ± 900 kg^−1^, respectively) compared with March 2019 and October 2019 (700 ± 700 and 600 ± 500 kg^−1^, respectively) which contained significantly more plastic per kilogram than the remaining sediment samples.

Almost all the 696 items analysed by FTIR were plastic (87%) (electronic supplementary material, fig. S2). The most common polymers were polypropylene (36%), polyester (17%), cellulosic items (11%), polyethylene (9%), acrylic (9%) and polyamide (7%). Cellulosic items included various semi-synthetic materials (rayon, viscose, avicel, azlon) bamboo, cellulose, cotton, linen and paper. The proportion of each polymer did not vary significantly between sampling dates.

### (d) Biota

In total 367 individuals of *Crangon crangon* (brown shrimp), 202 *Osmerus eperlanus* (European smelt), 258 *Platichthys flesus* (flounder) and 187 *Solea solea* (Dover sole) were collected. Over a quarter (27%) of *C. crangon* contained plastic, with an average of 0.39 ± 0.95 items individual^−1^. Nearly half (45%) of *O. eperlanus* contained plastic, with an average of 0.96 ± 1.43 items individual^−1^. Two thirds (65%) of *P. flesus* contained plastic, with 2.39 ± 3.43 items individual^−1^. For *S. solea*, 44% contained plastic, with 1.35 ± 2.78 items individual^−1^. The proportion of individuals contaminated in the gills and gastrointestinal tract is reported in the electronic supplementary material, table S5.

Model 2 showed that the proportion of individuals containing plastics was significantly different between sampling dates (*p* < 0.05) with the greatest proportion of contaminated individuals in December 2018 (winter) compared with March (spring) and June (summer) 2019 which were also significantly higher than all other dates (figure 3B). The proportion of individuals containing plastics was also significantly different between species. For combined contamination of gills and digestive tracts, fish were more contaminated than shrimp. When isolating ingested plastics, the benthic flatfish (*P. flesus* and *S. solea*) were more contaminated than *O. eperlanus* and *C. crangon*, with 49%, 33%, 28% and 27% of individuals contaminated, respectively. Throughout the sampling campaign, over 40% of *P. flesus* contained plastics with a low of 42% in September 2020 (autumn) and a high of 96% in December 2018 (winter). Greater variation in the proportion of animals to contain plastic was seen all other fish species, with between 9% (summer; July 2020) and 81% (summer; June 2019) of *S. solea* containing plastics. In March 2020 (spring) 13% of *O. eperlanus* contained plastics compared with 100% in March 2019 (spring). A lower proportion of *C. crangon* contained plastic across dates, with between 8% and 34% of individuals contaminated in June 2019 (summer) and September 2020 (autumn), respectively.

**Figure 5.**
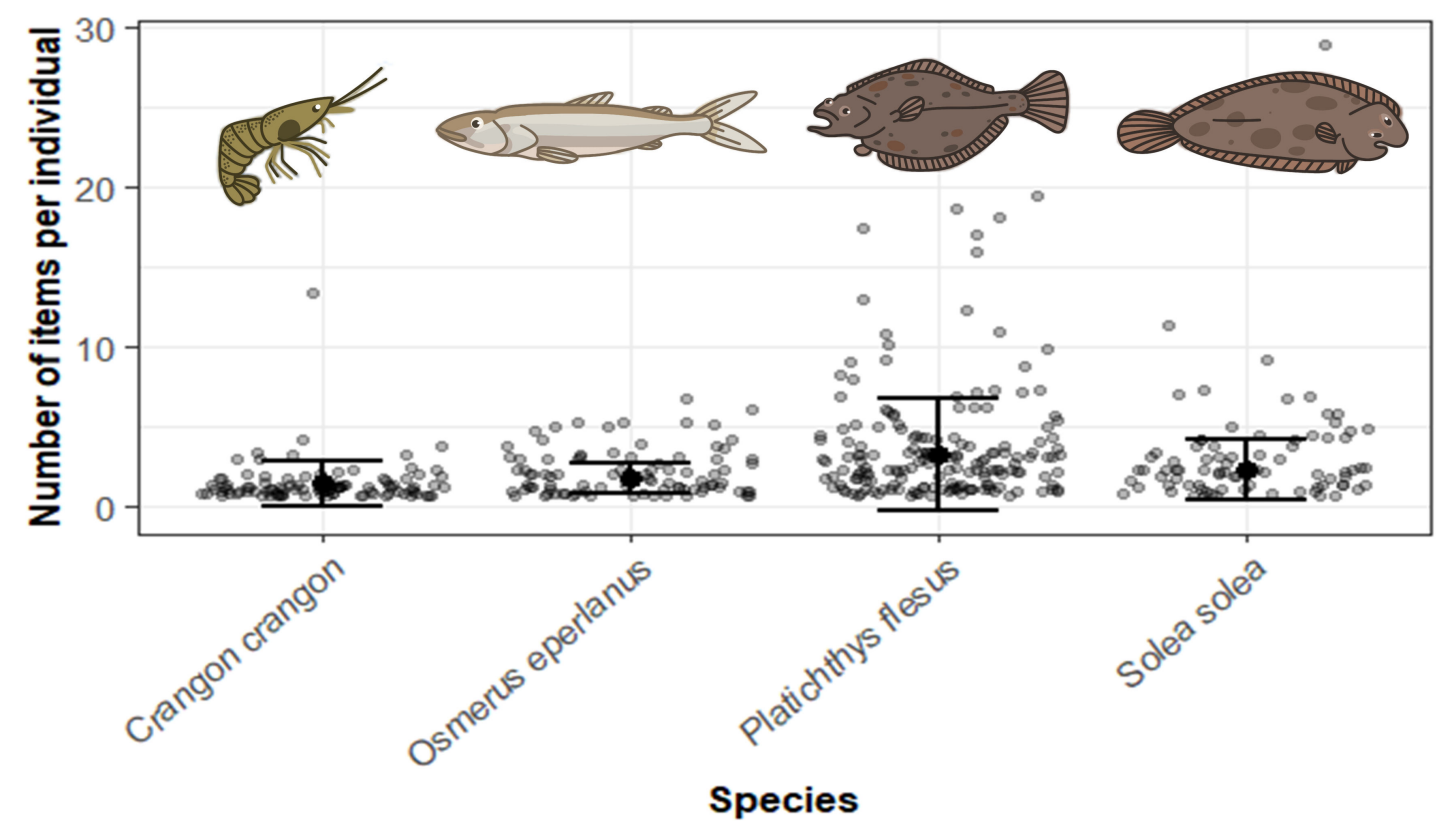
Plastic ingestion by biota varied significantly between species. Grey points are individual values, black points are the means and bars are ± s.d.

Model 3 found that length and mass of biota were not statistically significant and they were therefore removed from the model. As with model 2, sampling date and species were both statistically significant (*p* < 0.05). On average, 4.1 ± 4.3 items individual^−1^ were present in biota in June 2019 (summer) which was significantly higher than on all other sampling dates. *Platichthys flesus* was the most contaminated species with 3.2 ± 3.5 items individual^−1^ (average and s.d. after zeros were removed for model), followed by *S. solea* which contained 2.3 ± 1.9 items individual^−1^ (figure 5). *Osmerus eperlanus* and *C. crangon* contained the least plastics with 1.7 ± 0.9 items individual^−1^ and 1.4 ± 1.4 items individual^−1^, respectively.

For all species, blue and clear fibres were the most abundant form of plastic. Both were dominant in approximately half of *P. flesus* samples, with blue fibres most common in four of seven dates for *S. solea*. For the remaining three sampling dates, clear, red and purple fibres were abundant. Regardless of the sampling date, blue fibres were dominant in *O. eperlanus* and *C. crangon*. For *C. crangon* red fibres were equally prominent in October 2019 (autumn) and purple fibres were also abundant in June 2019 (summer). *Platichthys flesus* contained plastic between 60 µm and 77.9 mm long (mean ± s.d. 4.2 ± 6.4 mm; electronic supplementary material, table S5), while *S. solea* contained items between 67 µm and 62.3 mm long (3.1 ± 6.0 mm). Particles between 32 µm and 37.6 mm (1.8 ± 3.7 mm) were present in *O. eperlanus* and *C. crangon* contained plastics between 75 µm and 7.8 mm long (1.4 ± 1.4 mm). *Platichthys flesus* and *S. solea* ingested the most mesoplastics, with 26% and 16%, respectively (electronic supplementary material, fig. S1).

FTIR analysis was conducted on 82 items from *O. eperlanus*, 131 items from *P. flesus*, 68 items from *S. solea* and no items from *C. crangon* (due to time constraints). Most items in biota were cellulosic (*O. eperlanus*: 77% of items, *P. flesus*: 37%, *S. solea*: 54%, electronic supplementary material, fig. S2). For *P. flesus* a greater proportion of plastic items were identified than cellulosic (63% of items). These were most commonly polyester (31%) and polypropylene (10%). Polyester and polypropylene were also the most common plastic polymers ingested by *O. eperlanus* (7% each) and *S. solea* (14% and 12%, respectively). Other polymers present included acrylic, polyethylene, polyamide, polybutylene, epoxy resin and mixed polymers.

### (e) Rainfall

Shapiro–Wilk tests revealed that no variables were normally distributed (*p* > 0.05) and as such a non-parametric Spearman’s rank test was performed. Plastic abundance in sediment was not correlated with rainfall (*p* > 0.05). Contamination in biota, however, had a significant weak positive correlation to rainfall (*p* = 0.0007, *ρ* = 0.1061). Plastic diversity in sediment was not correlated for either cumulative rainfall or rainfall on the day prior to sampling. Histograms and scatter plots are available in the electronic supplementary material.

## 4. Discussion

### (a) Global context to meso/microplastic abundance

Plastic concentrations in riverine water can fluctuate greatly and sediment has been proposed as a more stable matrix to assess [53]. Many studies have explored concentration of riverine microplastic using sediment [42,69–86]. In a global setting, the concentrations of plastic reported in the present study are not particularly high but do exceed estimates for numerous other rivers. Typically, riverine sediment in Europe, such as the Thames, is more contaminated than that in Asia, Africa and the Americas, while the opposite is true for water samples [30]. The highest concentrations of microplastics in riverine sediment in Europe have been reported in the River Rhine, Germany (11 070 items kg^-1^) [72]; but the same study also reported some stations with concentrations far lower than the present study (260 kg^-1^). Most recovered items were far smaller than those detected in the present study (11–500 µm), with small microplastics generally being more abundant in the environment. Klein *et al.* [70] also reported that small microplastics (63–200 µm) were most abundant in the Rhine, but only recovered 1077 items kg^-1^, in line with the present study. The urban canals of Amsterdam [71] and the Spanish Ebro River [69] were also more contaminated than the present study but are still comparable. It is likely that in many rivers, flow rates flush plastic out to sea rather than accumulating in the sediment. In the case of the Ebro River [69], it is suspected that as a salt wedge estuary, the hydrodynamics facilitate microplastic sinking. In these estuaries, saltwater sits below freshwater which is rapidly flowing over the top. This promotes sedimentation at the salinity front [36]. The Thames Estuary, however, experiences strong vertical mixing and is only slightly stratified [87]. Vermeiren *et al.* [36] suggest that partially mixed estuaries, such as the Thames, are a greater source of microplastics to the sea, but acknowledge the lack of available evidence. Indeed, microplastics in the surface waters of the Thames are abundant [49], but Erith is a region of high sedimentation consisting of large mud flats. This could be one explanation for the relatively abundance of plastics in sediment in the present study.

It is surprising that despite the high population density of Chinese cities, higher concentrations of microplastics were recorded in the present study compared with the Beijiang River [78], a tributary of the Pearl River. It passes through Qingyuan City which has a population of over 4 million with 18 wastewater treatment plants discharging into the river. Given that this is just part of the 52 068 km^2^ Beijiang catchment and compared with the Thames watershed of 16 000 km^2^ encompassing 15 million residents [54], it raises concerns for the river’s health. Indeed, Belize has a small population with only 61 000 residents in Belize City, through which the Belize river flows. Yet, high concentrations of microplastics are recorded in its sediment [84]. This could be due to poor waste management and a lack of recycling facilities [88]. While a high population density is not always correlated with microplastic abundance it could be a contributing factor in the Rhine which supports a much larger population of *ca* 60 million [55]. High microplastic concentrations were also recorded in the Ebro River, Spain which only has a population of 6.7 million within its catchment [69]. The sewage system servicing London was antiquated and not designed to support the capital’s modern population. London is now supported by a ‘super sewer’ in the form of the Thames Tideway Tunnel, recently commissioned for use, the river was previously, at the time of the present study, at risk from pollution. This is exacerbated with sewage release from combined sewer outfalls. It is likely that untreated sewage is a major source of microplastics in the river. Certainly, concentrations in other European waterways are comparable with wastewater outfalls [71] and vegetation in rivers can cause large items to tangle and accumulate [48,89] providing ample opportunity for fragmentation.

While relatively high in a global context, compared with previous studies in the Thames, the present study reports concentrations in line with previous estimates. Certainly, estimates of microplastic abundance in the Thames surface water was higher than many other rivers globally [49]. For sediment analysis, the present study utilized a smaller mesh size yet still reported an abundance of similar sized plastic items as previous studies (i.e. 1−2 mm) [42]. A greater concentration of plastic was, however, recovered in the present study, with 71% of measured items in sediment being larger than 1 mm. In the non-tidal tributaries of the Thames, Horton *et al.* [42] reported an average concentration of between 185 and 332 items kg^−1^ whereas the present study is an order of magnitude higher. While small plastic items are abundant, the mean particle size of the present study was comparable with Horton *et al.* [42] and therefore size is not the only explanation for the observed difference. It is possible that plastic inputs have increased between 2014 and 2020 or that plastic is slowly accumulating in the river sediment. The tidal reaches of the estuary also receive inputs from these tributaries and are therefore collecting microplastics from more sources. The polymers reported in the present study are similar to those in previous studies [42,90].

A global review of microplastic ingestion by fish concluded that on average 50% of individuals contain microplastics, typically 3.5 items per fish [91]. This value is in line with the results of the present study. The review also noted that detritivores ingest more microplastics [91] which is again true for the present study. For *C. crangon*, similar levels of ingestion have been reported in other carideans, including the Indian prawn, *Fenneropenaeus indicus* in India (0.39 ± 0.6 per individual) [92] and the Australian glass shrimp, *Paratya australiensis* in Australia (0.52 ± 0.55) [93]. Slightly higher estimates have been recorded in *C. crangon* in Belgium (1.23 ± 0.99) [94] and the red prawn, *Aristeus antennatus* from the Mediterranean Sea (1.66 ± 0.11) [95], while estimates of up to 7.8 items per individual have been reported in Bangladesh [96] and the Persian Gulf [97]. In China, shrimp have been reported to contain nearly 30 items per individual [78].

Plastic contamination of biota in the Thames is typically to the same degree as the global average estimated by Wootton *et al.* [91]. The first recorded evidence of microplastic ingestion in the Thames found that between 20% and 75% of fish contained microplastics with an average of 0.2−0.85 items per individual [45]. Later studies reported a lower proportion of contaminated individuals (14−33%) but a higher average contamination (1.5−3.2 items per fish) [46]. Similarly, Horton *et al.* [44] reported microplastic ingestion of between 1.5 and 3 items per individual (flounder 1.98 microplastics per fish, whiting 2.46 microplastics per fish, herring 1.47 microplastics per fish). As in the present study, *C. crangon* tend to be less contaminated than fish with fewer items per individual and fewer contaminated individuals [46]. Certainly, compared with other decapods, the contamination levels of *C. crangon* in the present study are considerably lower than other estimates in the UK. Almost all brachyuran crabs from the Thames ingested microplastics, some with over 100 items in their gastric mill [47] despite being sampled at the same time and location as the shrimp in the present study.

All plastic collected from biota was in a similar size range to that in the sediment with average particle size being comparable with sediment in *C. crangon* and *O. eperlanus*. Whereas *P. flesus* and *S. solea* tended to eat larger plastic items. While clear and white fibres dominated sediment samples, blue fibres were more abundant in biota digestive tracts. In addition, the fibres in the sediment tended to be polypropylene and polyester, whereas fish and shrimp ingested cellulosic fibres. Clear fibres were slightly more abundant in the diet of *P. flesus* and *S. solea*, indicating that demersal organisms are still exposed to the plastics in the riverbed. The abundance of blue fibres might be explained by preferential predation as has been reported in some planktivorous fish [98,99]. This preference might then be magnified up the food web. Alternatively, blue particles might be more abundant in the water. In the present study, the gills were used as a proxy for what was in the water and blue fibres were indeed abundant. Due to potential contamination, fibres have been excluded from previous investigations of microplastic abundance in the Thames Estuary water [49], which limits validation with environmental data. More recent studies by Devereux *et al.* [51,52] did include fibres and reported that black fibres were most abundant. Both Rowley *et al.* [49] and Devereux *et al.* [51,52] noted that plastic polymers (polypropylene and polyethylene, and polychloroprene and polyvinyl chloride, respectively) were more abundant in the water than the cellulosic items which are primarily recovered from biota in the present study. Both studies also recovered large amounts of glitter which were not observed in biota or sediment. These studies, however, investigated surface water concentrations and blue fibres may indeed be more abundant lower down the water column. Certainly, Nan *et al.* [93] concluded that shrimp in Australia directly consumed microplastics from the water.

### (b) Seasonal variation in plastic abundance

With discussions around how to ensure sample collection is representative of the environment, seasonal sampling can address some of these concerns [53]. Seasonal variation in meso- and microplastic abundance in the sediment reflected that of macroplastics in the estuary with peaks in June (summer) and September 2020 (autumn) [48] after periods of short, heavy rainfall. Urban centres are major inputs of plastics for rivers, with various sources within them. Therefore, runoff from cities on the Thames, such as London, could be the cause for the greater diversity of plastic in these months. Indeed, in South Korea sites near urban centres had a greater diversity of plastic than at sites near aquaculture or rural areas [81,100]. Heavy rain was recorded in June and August, probably contributing to the increased abundance of microplastics. For macroplastics, wet wipes and sanitary pads which are associated with sewage outfalls were more abundant following heavy rain [47], further supporting antiquated sewage systems as a significant plastic input in the Thames. The most common items in sediment were white/clear fibres which may originate from wipes and sanitary products. The most common polymers in the estuary, polypropylene and polyester, are also commonly used in these products including those recovered from the Thames foreshore [50]. Furthermore, spring tides, such as those in July (summer) and September 2020 (autumn), cause less vertical mixing than neap tides [101] and thereby allow for the preferential deposition of microplastics. This probably contributed to the samples collected at these times being significantly more contaminated than other samples.

After periods of longer, lighter rainfall plastic abundance in sediment was lower. Prolonged rain can result in higher flow rates in estuaries which can flush pollutants away from hotspots, such as outfalls [102]. In addition, rainfall can create hydrodynamic forces that prevent plastic deposition in sediment [77]. Certainly, when there is no rain plastics are stored in the sediment [103]. The Thames estuary is naturally turbid, with strong vertical mixing [56] and rainfall increases the ebb velocities, flushing out sediment. This could explain why concentrations in the sediment were lower in December 2018 (winter), June 2019 (summer) and December 2019 (winter).

The majority of studied species are benthic and therefore plastic in the sediment is still bioavailable to them. Accumulation in sediment, however, does not coincide with abundance in biota. Peaks in ingestion seem to occur in the sampling trip following peaks in plastic accumulation in sediment. The most notable instance of this is observed in June 2019 (summer) following observed elevation in plastic abundance in sediment in March 2019 (spring). The same trend is seen in October (autumn) and December 2019 (winter). It is possible that there is a delay between plastic entering the estuary, accumulating in the sediment and being disturbed or resuspended from the sediment and ingested. As flatfish species, *P. flesus* and *S. solea* live on the riverbed and disturb the sediment surface when moving or feeding, they are more likely to ingest plastics when concentrations in the sediment are higher. Plastic ingestion is, however, reported to vary with surveys conducted a year apart estimating significantly differently proportions of fish to ingest plastic and average plastic abundance [45,46]. In June 2019 (summer), prior to sampling was the greatest cumulative rainfall over the 2 yr study period. This probably led to resuspension of sediment during vertical mixing, making plastics easier to passively ingest by all species. Similar trends with rainfall have been observed in shrimp from India [92] and the North Sea [94] and some fish species in Brazil [104] . Both Silva *et al.* [104] and Ferreira *et al.* [105,106] noted that prey varied seasonally and that this probably affected the likelihood of some species ingesting plastics.

Biological factors may also influence seasonal variation, with evidence that seasonal prey variation affects microplastic exposure in some fish species [104–106]. Certainly, organisms with multiple feeding strategies experience different plastic exposures as they switch between feeding modes [107]. The diet of *O. eperlanus* also varies monthly and annually. In the Thames, *O. eperlanus* typically predates upon mysids; amphipods, primarily gammarids; *Crangon crangon*; small fish; fish eggs and copepods [108,109]. In the present study, *O. eperlanus* almost exclusively ingested amphipods (67% of individuals) and shrimp (46%), but seasonal variation in prey abundance was not investigated [59]. In the colder months of autumn and winter, mysids become more dominant in the diet, with gammarids more abundant in spring and summer [109]. The proportion of contaminated *O. eperlanus* does not match with this dietary change, with no consistent peak in ingestion at any particular season. Feeding intensity of *Osmerus eperlanus* in the Thames may change seasonally as they exhibit a varying condition index [109]. Many individuals do not survive after spawning in spring, around February to April. Indeed, the proportion of individuals contaminated in March 2020 was low at only 13%. All individuals were contaminated in March 2019, but a smaller sample size was collected at this time.

*Solea solea* feed at night, predating worms, molluscs and small crustaceans [110]. In 2015, fish from the Thames Estuary ingested *Crangon crangon* and other shrimp, some brachyuran crabs, bivalves and polychaetes [59]. Amphipods were present in 76% of fish stomachs in the present study. Sediment and polychaetes were the subsequent most commonly recovered items. Shrimp and fish were also present [59]. *Platichthys flesus* eat benthic fauna including small fish and invertebrates [111]. Fish collected in 2015 ingested amphipods, primarily *Corophium volutator*, polychaetes, mostly Nereididae, fish, bivalves and *Crangon crangon* [59]. During the present study, fish were found to have ingested primarily amphipods (in 45% of individuals), including *Corophium volutator*, shrimp, sediment and stones, polychaetes, *Crangon*, isopods, fish, bivalves, barnacles, gastropods and vegetation. This clearly demonstrates that plastic in the substrate is bioavailable to *P. flesus* [59]. Like *Osemerus eperlanus*, energy expenditure during spawning affects *P. flesus* seasonally. Growth rate reduces in winter after spawning with increased growth in summer [112]. Growth rate is strongly linked to feeding rate [113–115]. Indeed, invertebrate abundance is greatest in summer [112]. In the present study, the proportion of individuals to ingest plastic remained fairly constant around 40−45%, with peaks in December 2018 (winter) and June 2019 (summer). This does not consistently indicate increased feeding in summer leads to an increase in plastic ingestion. However, reduced activity in London and other urban centres during the COVID-19 lockdowns may have led to reduced terrestrial inputs of plastics during the summer of 2020. This could explain the lower proportion of contaminated individuals and reduced abundance of plastic in biota at this time.

Most of the stomach contents of *Crangon crangon* were unidentifiable. Individuals collected in 2015 by McGoran *et al.* [46] contained polychaetes and amphipods. Individuals collected for the present study also contained polychaetes and amphipods, with some sediment also present. This aligns with other studies that reported that the species primarily feeds on polychaetes, followed by bivalves and crustaceans [116]. The authors also noted that the shrimp did not move to find preferred prey but rather fed on what was abundant in that region of the estuary. *Crangon crangon* probably remain in the estuary all year, even during spawning seasons in autumn [116]. With a benthic lifestyle, it is likely that *Crangon crangon* will be influenced by any seasonal variation in sediment plastic accumulation. Indeed, the highest proportion of contaminated shrimp was in September 2020 (autumn), July 2020 (summer) and December 2019 (winter). The former two samples align with peaks in sediment concentrations. The latter peak in ingestion, however, occurred when sediment concentrations were low. As such, other variables probably affect plastic bioavailability. Rainfall is a key factor in increasing plastic inputs to riverine systems and has been linked to increased microplastic ingestion in shrimp [92,94]. In the present study, ingestion had a weak positive correlation with rainfall but was not correlated with sediment. Some plastic probably stays suspended in the water column and can be ingested by some species. Fast flowing water from rainfall may reduce bioavailability for other species.

In March 2020 (spring), London went into lockdown as part of precautions against the COVID-19 pandemic. The samples collected in March 2020 (spring) were a few days prior to the formal lockdown, allowing for a true ‘pre-event’ sample collection. Following the lockdown, concentrations of plastics in the sediment increased. A similar trend has also been reported in surface water samples from the River Thames [38]. There was, however, no observed increase in plastics in biota following the COVID-19 London lockdown. Due to the three-month break between sampling dates, a true ‘post-event’ concentration could not be collected and therefore it is difficult to truly estimate the effect of the pandemic on plastic abundance in the estuary.

### (c) Limitations of the present study

Plastic containers were used during sample collection. These were washed prior to use to remove any contamination within. Containers were covered until use and thereafter moved as little as possible to minimize any abrasion from the sediment. As contamination blanks were not collected in the field it is only assumed that contamination was prevented at this stage. In addition, nylon (PA) filters were used. Several nylon filters were examined under the microscope before sample processing began. When fibres shed from these filters, they retained a characteristic wave due to being woven into the filter. This was easily identifiable and no fibres with this shape were counted in the analysis. In addition, PA only accounted for 7% of plastic in sediment and was also uncommon in biota samples.

In the present study, a 32 μm mesh size was used to extract meso- and microplastics. This may have resulted in the underestimation of microplastic abundance as small items would not have been detected. Under the newly established OSPAR indicator for monitoring microplastics in seafloor sediments [29] it is necessary to report items down to 100 μm in the longest dimension, which this study meets. Items down to 50 μm or 20 μm can be reported with the aid of automated identification techniques such as focal plane array FTIR or laser direct infrared analysis. In the present study, all plastics in sediment were larger than 100 μm, whilst biota contained smaller items down to 32 μm. Electronic supplementary material, table S4, however, highlights that the most common mesh size for microplastic extraction from estuarine or riverine sediment is less than or equal to 5 μm (*ca* 52% of compared studies). Mesh sizes of 20 μm, 30 μm and 63 μm are also common, with some studies using large mesh sizes of 300 μm or 1 mm. Mani *et al.* [72] reported that 96% of microplastics in the River Rhine, Germany were smaller than 75 μm (but did not report microplastics larger than 500 μm). The concentrations reported in this study of the Rhine were the highest globally, being an order of magnitude higher than those reported in the present study. Mani *et al.* [72] used a 0.2 μm filter to capture all detectable particles. Leslie *et al.* [71] and Simon-Sánchez *et al.* [69] also both used filters with pores smaller than 1 μm and recorded concentrations greater than that of the present study. Yet, in these studies particles larger than 300 μm [71] and 200 μm were most abundant [69]. While riverine discharge to the sea is high in Asian rivers, considering microplastic concentrations in surface water samples [30], concentrations in the sediments of Asian estuaries were generally lower than the present study, despite studies using smaller filters [73–79]. Rao *et al.* [73] reported that only 14% of particles were smaller than 200 μm despite using a 32 μm filter; however, this could also be due to the lack of smaller particles in the samples. Many studies from Asia do not report microplastic concentrations by size fractions and thus it is not clear how abundant smaller particles are in these estuaries. In Canada, the St. Lawrence River was dominated by microbeads which led to an abundance of microplastics of less than 400 μm (between 0−98% of items in samples) [83]. In the River Thames, Rowley *et al.* [49] detected microbeads in almost all samples despite using a 250 μm plankton net and a 32 μm mesh during sample collection and processing, respectively. Out of 5557 plastics visually identified in the present study only seven were microbeads. Therefore, Rowley *et al.* [49] and the authors of the present study probably underestimated smaller particles. Aside from mesh size, the sample collection methods and processing methods of most estuarine sediment studies are similar (electronic supplementary material, S4) and do not explain the differences in recorded abundance of plastics.

## 5. Conclusion

Meso- and microplastic abundance vary seasonally, both in the sediment and in biota, but do not peak at the same time as each other. Plastic presence in biota at different trophic levels (shrimp and fish) raises concerns for ecosystem health in the Thames. As complex systems, many factors affect plastic distribution in estuaries but data on a finer scale resolution, such as monthly sampling with ad hoc sampling after key weather events such as storms, are needed to unpick the key drivers of plastic retention, ingestion and flushing seasonally. Water samples should be collected in conjunction with sediment and biota to better represent plastics flushed downstream and potentially out to sea. In addition, the implementation of longer-term studies could also evidence the causation of plastic fluxes, being able to include a greater diversity of environmental conditions such as extreme weather events which do not occur annually. With the development of a modern sewage system for London scheduled for 2025, long-term studies can also be beneficial in recording the success of such mitigation measures. While environmental data are needed to measure the success of policy and mitigation measures and to inform ecotoxicological studies, the development of risk assessments is needed to better inform future decisions. The present study provides evidence that a wide diversity of plastic and semi-synthetic items, most commonly fibres, are ingested by biota. These should be included in ecotoxicological assessments to determine the effect of mixed contaminants on biota rather than single polymer or shape tests.

## Data Availability

Microplastic raw data are available in Figshare [117]. Supplementary material is available online [118].
